# The complete chloroplast genome of *Primula amethystina* subsp*. argutidens* (Primulaceae)

**DOI:** 10.1080/23802359.2023.2231108

**Published:** 2023-07-06

**Authors:** Shu-Bao Wang, Yun-Qi Liu, Li Zhang, Rui Li, Yuan Huang

**Affiliations:** School of Life Sciences, Yunnan Normal University, Kunming, PR China

**Keywords:** Chloroplast genome, *Primula amethystina* subsp. *argutidens*, phylogenetic analysis

## Abstract

*Primula amethystina* subsp. *argutidens* (Franchet) W. W. Smith & H. R. Fletcher (1942) is a blooming plant of the family Primulaceae. Here, we sequenced, assembled, and annotated the complete chloroplast (cp) genome of *P. amethystina* subsp. *argutidens*. The cp genome of *P. amethystina* subsp. *argutidens* is 151,560 bp in length with a GC content of 37%. The assembled genome has a typical quadripartite structure, containing a large single-copy (LSC) region of 83,516 bp, a small single-copy (SSC) region of 17,692 bp, and a pair of inverted repeat (IR) regions of 25,176 bp. The cp genome contains 115 unique genes, including 81 protein-coding genes, four rRNA genes, and 30 tRNA genes. Phylogenetic analysis showed that *P. amethystina* subsp. *argutidens* was closely related to *P. amethystina*.

## Introduction

**Figure 1.**
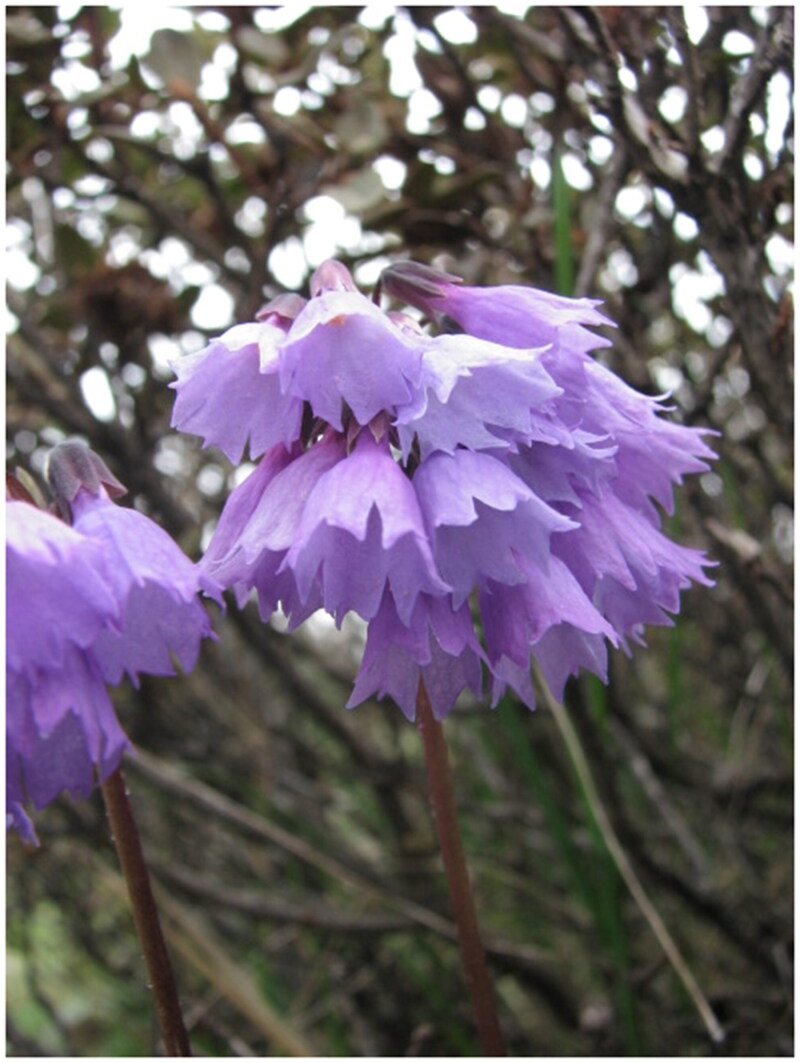
*P. amethystina* subsp. *argutidens* species reference image was taken in Shangri-la, Yunan Province, China (27°37′11″N, 99°38′29″E) and provided by corresponding author Huang Yuan. It is a perennial herb with rich violet-blue flowers and deeply serrated leaves.

*Primula amethystina* subsp. *argutidens* (Franchet) W. W. Smith & H. R. Fletcher (Smith and Fletcher 1942) is a blooming plant of the family Primulaceae (Figure 1). In Flora of China, *P. amethystina* subsp. *argutidens* is classified as a subspecies of *P. amethystina* (Hu and Kelso 1996). This subspecies is a perennial herb with rich violet-blue flowers and deeply serrated leaves, mainly distributed in alpine grasslands at an altitude of 3500–5000 m in the western Sichuan Province of China (Hu and Kelso 1996). Compared with *P. amethystina* and *P. amethystina* subsp. *brevifolia*, *P. amethystina* subsp. *argutidens* has a more petite stem body, deeper-toothed leaf margins, shorter pedicels, and a profoundly recessed corolla lobe end (Hu and Kelso 1996). Although cp genome is widely used in phylogenetic research and molecular identification in many families of angiosperm, plastome phylogenomic studies of the phylogenetic relationship between *P. amethystina* subsp. *argutidens* and *P. amethystina* are lacking (Huang et al. 2017; Bi et al. 2018). Here, we report the first complete cp genome of *Primula amethystina* subsp. *argutidens* to provide a plastome database for further phylogenetic analysis.

## Materials and methods

In this study, *P. amethystina* subsp. *argutidens* was sampled from Shangri-la of Yunnan Province, China (27°37′11″N, 99°38′29″E). Voucher specimens (HY-22) were deposited in the Herbarium of Yunnan Normal University (YNUB, Website: https://life.ynnu.edu.cn/, Contact: Jian-Lin Hang, Email: hjlynub@163.com). Total genomic DNA was extracted using a modified CTAB method (Porebski et al. 1997). The fragmented genomic DNA was used to construct short-insert libraries for Illumina paired-end (PE) sequencing on the Illumina Hiseq X Ten sequencer. We obtained 17,593,572 filtered reads and then assembled the plastid genome using the software NOVOPlasty v2.7.2 (Dierckxsens et al. 2017) with *P. amethystina* as the reference genome (GenBank accession no. NC_053577). Then, we used Geneious V2020.1.1 software (Kearse et al. 2012) to annotate the cp genome of *P. amethystina* subsp. *argutidens* using *P. amethystina* (Wang et al. 2021) as a reference and then used *Primula bulleyana* (Chen, Zhang et al. 2019) as a reference for modification and correction. The annotated cp genome sequences of *P. amethystina* subsp. *argutidens* were deposited in the GenBank database under the accession no. ON416902. The circular *Primula amethystina* subsp. *argutidens* genomic map and schematic map of the cis and trans splicing genes were drawn using CPGView (Liu et al. 2023, http://www.1kmpg.cn/cpgview/). To verify the accuracy of the assembly, we mapped clean reads to the assembled chloroplast (cp) genomes to assess the depth of coverage (Figure S1). We used DnaSP6.12 to perform a sliding window analysis of nucleotide diversity to evaluate the divergence of the four *Sect. Amethystina* cp genome sequences (Librado and Rozas 2009).

In order to further determine the phylogenetic relationship of *P. amethystina* subsp. *argutidens* within the genus *Primula*, the multiple sequence alignment of the complete cp DNA of 47 species from the Primulaceae was analyzed by MAFFT (Katoh and Standley 2013). Four *Lysimachia* species, four *Androsace* species, and *Glaux maritima* were chosen as outgroups for constructing a phylogenetic tree. The maximum-likelihood (ML) tree was constructed using IQ-TREE v1.6.10 (Nguyen et al. 2015), and the best-fit model according to the Bayesian information criterion (BIC) was TVM + F + R3 (Kalyaanamoorthy et al. 2017). Branch supports were tested using ultrafast bootstrap (UFBoot) (Hoang et al. 2018) and SH-like approximate likelihood ratio test (SH-aLRT) (Guindon et al. 2010) with 10,000 replicates.

## Results

**Figure 2.**
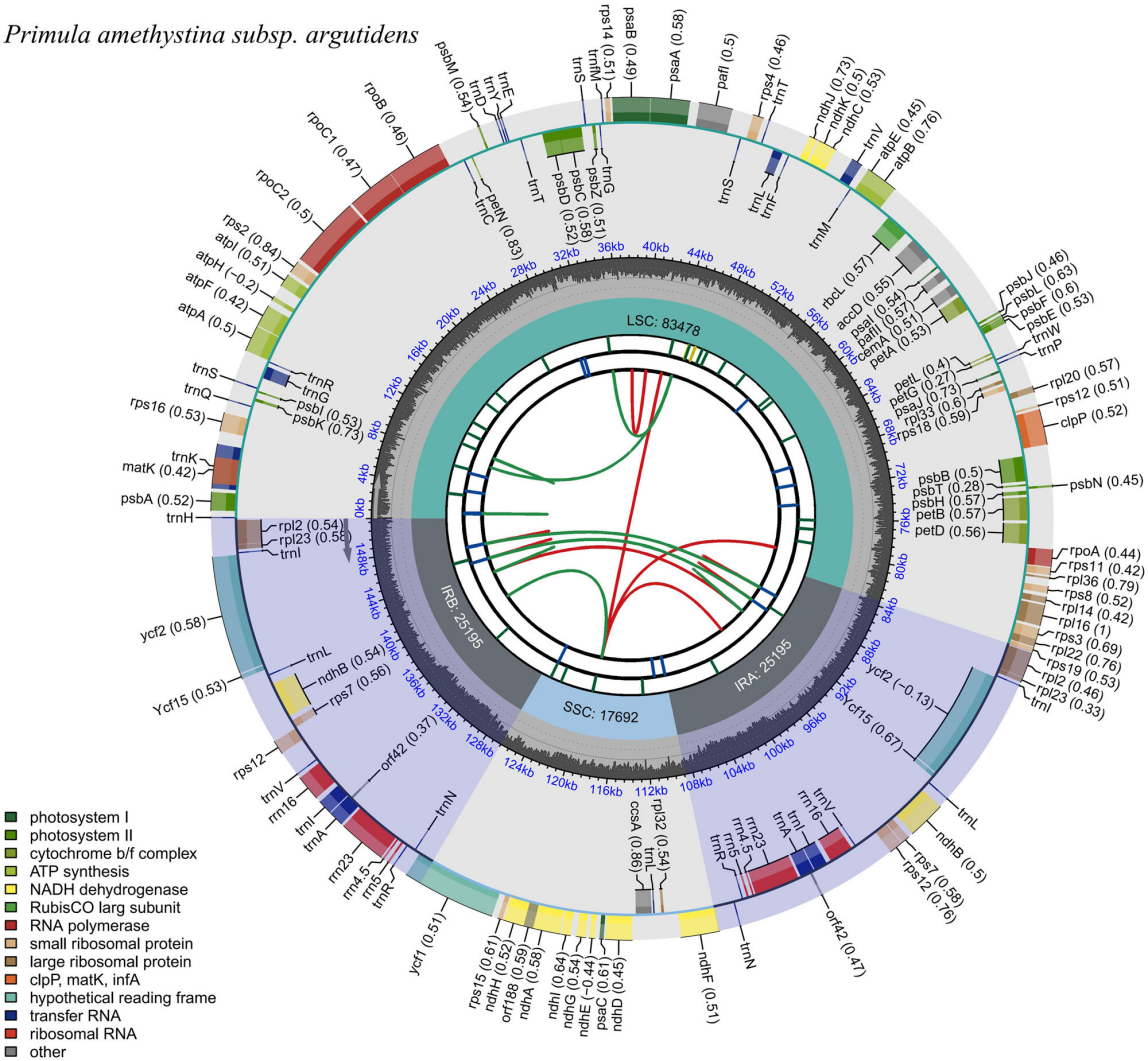
Genomic map of overall features of *Primula amethystina* subsp. *argutidens* chloroplast genome, generated by CPGview (http://www.1kmpg.cn/cpgview/). The species’ name is shown in the top left corner. The map contains six tracks by default. From the center outward, the first track shows the dispersed repeats. The dispersed repeats consist of direct (D) and palindromic (P) repeats, connected with red and green arcs. The second track shows the long tandem repeats as short blue bars. The third track shows the short tandem repeats or microsatellite sequences as short bars with different colors. The colors, the type of repeat they represent, and the description of the repeat types are as follows. The small single-copy (SSC), inverted repeat (IRa and IRb), and large single-copy (LSC) regions are shown on the fourth track. The GC content along the genome is plotted on the fifth track. The genes are shown on the sixth track. The optional codon usage bias is displayed in the parenthesis after the gene name. Genes are color-coded by their functional classification. The transcription directions for the inner and outer genes are clockwise and anticlockwise, respectively. The functional classification of the genes is shown in the bottom left corner.

**Figure 3.**
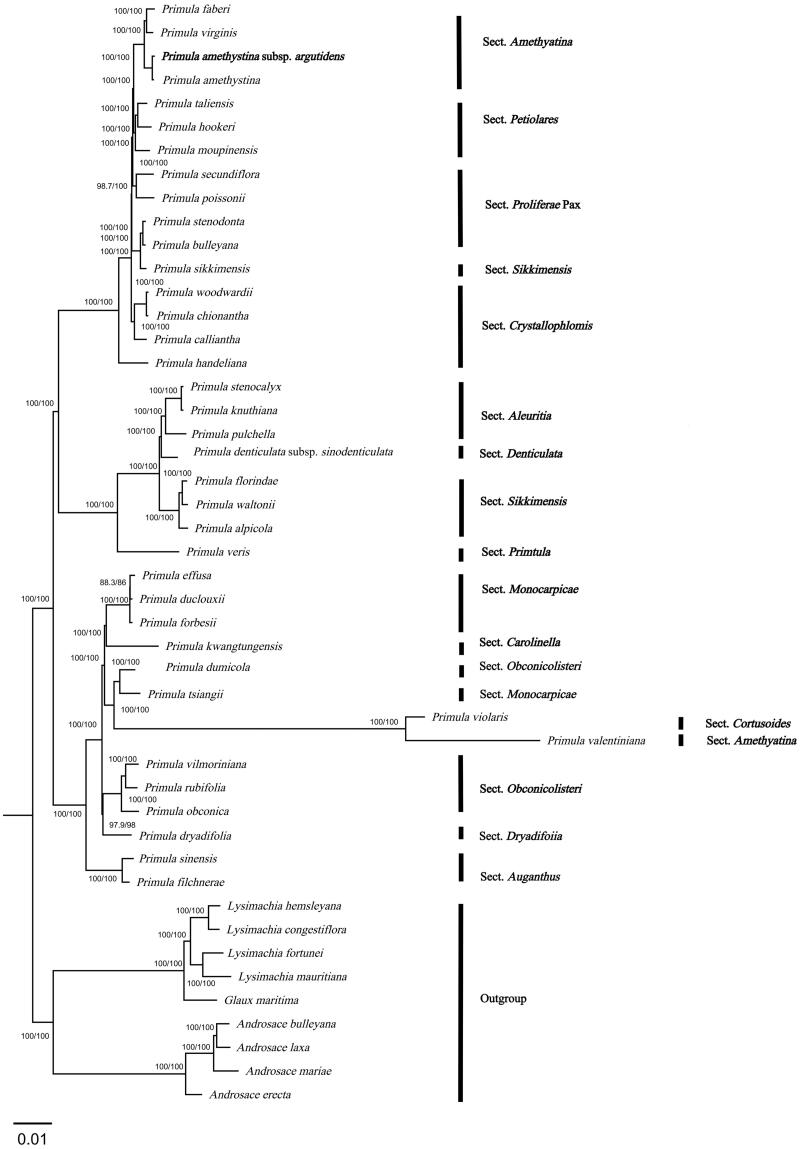
In the ML phylogenetic tree of *P. amethystina* subsp. *argutidens* and 46 Primulaceae species based on chloroplast genomes. The best-fit model according to the Bayesian information criterion (BIC) was TVM + F + R3. Branch supports were tested using ultrafast bootstrap (UFBoot) and SH-like approximate likelihood ratio test (SH-aLRT) with 10,000 replicates. *P. amethystina* subsp. *argutidens* is marked in bold. The following sequences were used: *Primula faberi* NC_053576 (Wang et al. 2021), *Primula virginis* NC_053581 (Wang et al. 2021), *Primula amethystina* NC_053577 (Wang et al. 2021), *Primula taliensis* NC_053601 (Wang et al. 2021), *Primula hookeri* NC_053593 (Wang et al. 2021), *Primula moupinensis* NC_050244, *Primula secundiflora* NC 053585 (Wang et al. 2021), *Primula poissonii* NC_024543 (Yang et al. 2014), *Primula stenodonta* NC 034677 (Zhang et al. 2017), *Primula bulleyana* NC_046947 (Chen, Zhang et al. 2019), *Primula sikkimensis* NC_050243, *Primula woodwardii* NC 039349 (Ren et al. 2018), *Primula chionantha* NC_053583 (Wang et al. 2021), *Primula calliantha* MZ054238 (Yang et al. 2021), *Primula handeliana* NC_039348 (Ren et al. 2018), *Primula stenocalyx* NC_058249 (Guo, Ma, Li et al. 2021), *Primula knuthiana* NC_039350 (Ren et al. 2018), *Primula pulchella* NC_050246 (Zhang et al. 2017), *Primula denticulata* subsp*. sinodenticulata* NC_050247, *Primula florindae* NC_053579 (Wang et al. 2021), *Primula waltonii* NC_058808, *Primula alpicola* NC_053588 (Wang et al. 2021), *Primula veris* NC_031428 (Zhou et al. 2016), *Primula effusa* NC_058259 (Zhong et al. 2019), *Primula duclouxii* NC_058263 (Zhong et al. 2019), *Primula forbesii* NC_061696, *Primula kwangtungensis* NC_034371 (Zhang et al. 2016), *Primula dumicola* NC_058257 (Zhong et al. 2019), *Primula tsiangii* NC_046755 (Chen, Yan et al. 2019), *Primula violaris* NC_050848 (Chai et al. 2021), *Primula valentiniana* NC_061669, *Primula vilmoriniana* NC_058258 (Zhong et al. 2019), *Primula rubifolia* NC_058261 (Zhong et al. 2019), *Primula obconica* NC_046415 (Zhang et al. 2019), *Primula dryadifolia* NC_053596 (Wang et al. 2021), *Primula sinensis* NC_030609 (Liu et al. 2016), *Primula filchnerae* NC_051972 (Lu et al. 2020), *Lysimachia hemsleyana* NC_052863 (Ying et al. 2019), *Lysimachia congestiflora* NC_045275 (Li et al. 2019), *Lysimachia fortunei* NC_052863, *Lysimachia mauritiana* NC_060700 (Lee et al. 2022), *Glaux maritima* NC_059901 (Liu et al. 2021), *Androsace bulleyana* NC_034641, *Androsace laxa* (Ren et al. 2018), *Androsace mariae* NC_051991 (Guo, Ma, Xie et al. 2021), and *Androsace erecta* NC 057637.

The complete cp genome of *P. amethystina* subsp. *argutidens* was 151,560 bp in length with an overall GC content of 37% and an average coverage of 1789.6 (Figure 2, Figure S1). The assembled genome has a typical quadripartite structure, containing a large single-copy (LSC) region of 83,516 bp and a small single-copy (SSC) region of 17,692 bp, which are separated by a pair of inverted repeat (IR) regions of 25,176 bp. The cp genome contains 134 genes, including 89 protein-coding genes, eight rRNA genes, and 37 tRNA genes, of which 81 CDS genes, four tRNA genes, and 30 rRNA genes are unique, respectively. Totally, 11 cis-splicing genes including *rps16*, *atpF*, *rpoC1*, *pafl*, *clpP*, *petB*, *petD*, *rpl16*, *rpl2*, *ndhB*, and *ndhA* (Figure S2A), and one trans-splicing genes *rps12* (Figure S2B) were detected. The cp genomes of four species of *Sect. Amethystina* were compared to screen single-nucleotide polymorphisms (SNPs) with higher nucleotide diversity value. As a result, the four most variable regions were *rpl2-trnH-GUG*, *trnH-GUG-psbA*, *trnK-UUU-rps16*, and *rpoB-trnC-GCA* of which three are located in the LSC region and one is in the SSC region. Partial sequences of *trnH-GUG*, *psbA*, *rps16*, *rpoB*, and *ycf1* also have rich variable sites (Table S1, Figure S3). The phylogenetic tree demonstrated that *P. amethystina* subsp. *argutidens* and *P. amethystina* formed a robust monophyletic clade, which was sister to each other (Figure 3).

## Conclusions

Our result supported well the subspecies of *P. amethystina* subsp. *argutidens.* The complete cp genome sequence of *P. amethystina* subsp. *argutidens* provided a valuable genomic database for further studies on the evolutionary history and phylogenetic relationship of *Primula* species.

## Supplementary Material

Supplemental Material

## Data Availability

The genome sequence data supporting the study’s findings are openly available in NCBI GenBank at https://www.ncbi.nlm.nih.gov/ under accession no. ON416902. The associated BioProject, SRA, and Bio-Sample numbers are PRJNA834988, SRR19175835, and SAMN28088488, respectively.
