# Supplementary material for: The complete chloroplast genome of Primula amethystina subsp. argutidens (Primulaceae)
Source: Mitochondrial DNA B Resour. 2023 Jul 6;8(7):737–41. doi: 10.1080/23802359.2023.2231108 (PMC10332233; doi:10.1080/23802359.2023.2231108)
Supplement: Supplemental Material [file TMDN_A_2231108_SM6070.docx]

**Content：**

**Table S1.** Detection of highly variable regions in four *Sect. Amethyatina* cp genomes

**Figure S1.** The coverage figure of the complete chloroplast genome of *Primula amethystina* subsp. *argutidens*.

**Figure S2.** Schematic map of the cis and trans splicing genes in the chloroplast genome of *Primula amethystina* subsp. *argutidens.*

**Figure S3.** Results of comparative chloroplast genomic analyses for four cp genome sequences of the *Sect. Amethyatina* species.

**Table S1**. Detection of highly variable regions in four *Sect. Amethyatina* cp genomes

| No. | Location in Sect. Amethyatina  cp genome (bp) | Mean nucleotide  diversity | Region | Highly variable regions in *P. amethystina* subsp*. argutidens* cp genome | | Location |
| --- | --- | --- | --- | --- | --- | --- |
| 1 | 1-1040 | 0.02433 | Region 1 | Section of IGS rpl2-trnH-GUG | (1−21 bp, length: 21 bp) | LSC |
|  |  |  | Region 2 | gene trnH-GUG | (22−96 bp, length: 75 bp) |  |
|  |  |  | Region 3 | IGS of trnH-GUG-psbA | (96−459 bp, length: 364 bp) |  |
|  |  |  | Region 4 | Tail of gene psbA | (460−1040 bp, length: 581 bp) |  |
| 2 | 4559-5572 | 0.01367 | Region 1 | Section of IGS trnK-UUU- rps16 | (4559−5081 bp, length: 523 bp) | LSC |
|  |  |  | Region 2 | Tail of gene rps16 | (5082−5572 bp, length: 491 bp) |  |
| 3 | 26290-27338 | 0.03283 | Region 1 | Head of gene rpoB | (26290−26455 bp, length: 166 bp) | LSC |
|  |  |  | Region 2 | Section of IGS rpoB-trnC-GCA | (26456−27338 bp, length: 883 bp) |  |
| 4 | 122928-123933 | 0.0115 | Region 1 | Section of gene ycf1 | (122928-123933 bp, length: 1005 bp) | SSC |
|  |  |  |  |  |  |  |


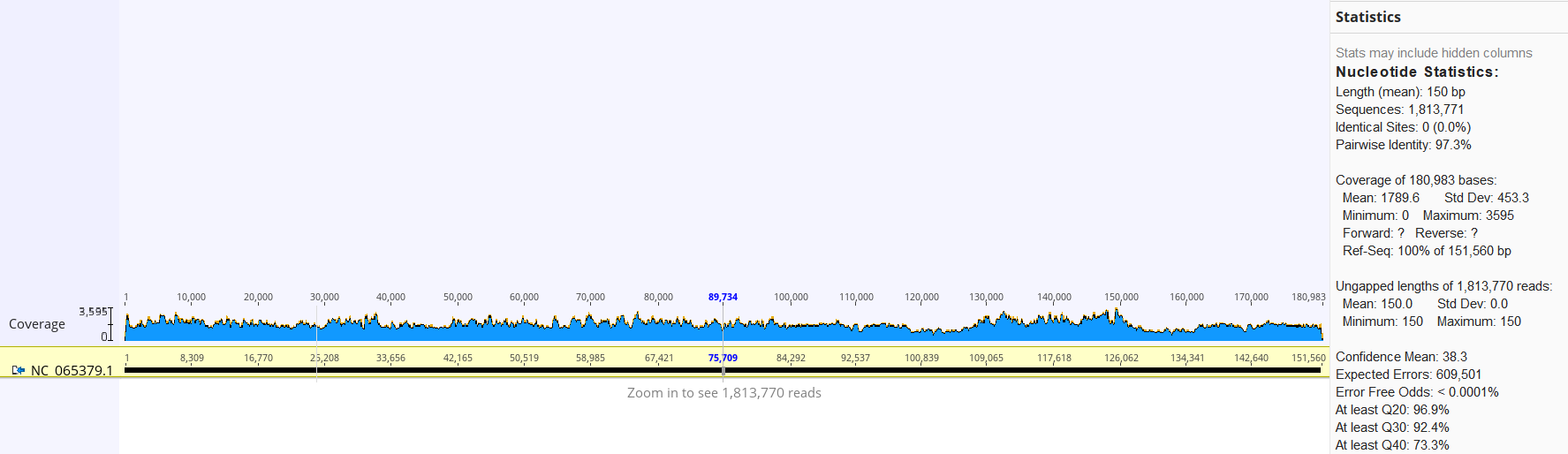
**Figure S1.** Overall coverage depth of the chloroplast genome assembly of *Primula amethystina* subsp. *argutidens*. This figure was generated using Geneious V2020.1.1 by aligning DNA-Seq data to the whole chloroplast genome. The height of the blue graph indicates the number of sequences at each location.


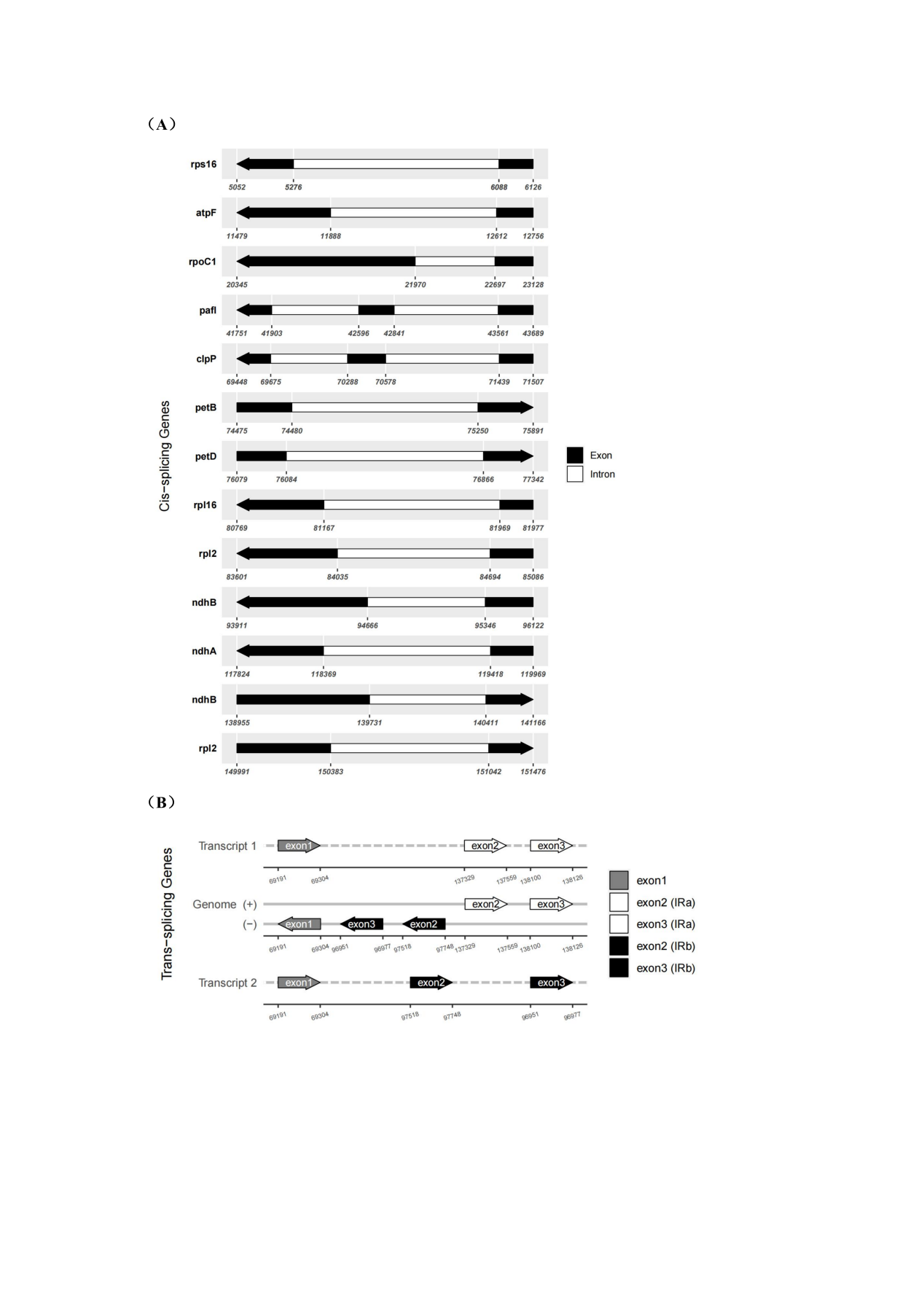
**Figure S2.** Schematic map of the cis-splicing genes (A) and trans-splicing gene rps12 (B) in the chloroplast genome of *Primula amethystina* subsp. *argutidens*. Exons are shown in black; Introns are shown in white. Arrows indicate the sense direction of genes. The map was generated using CPGview.


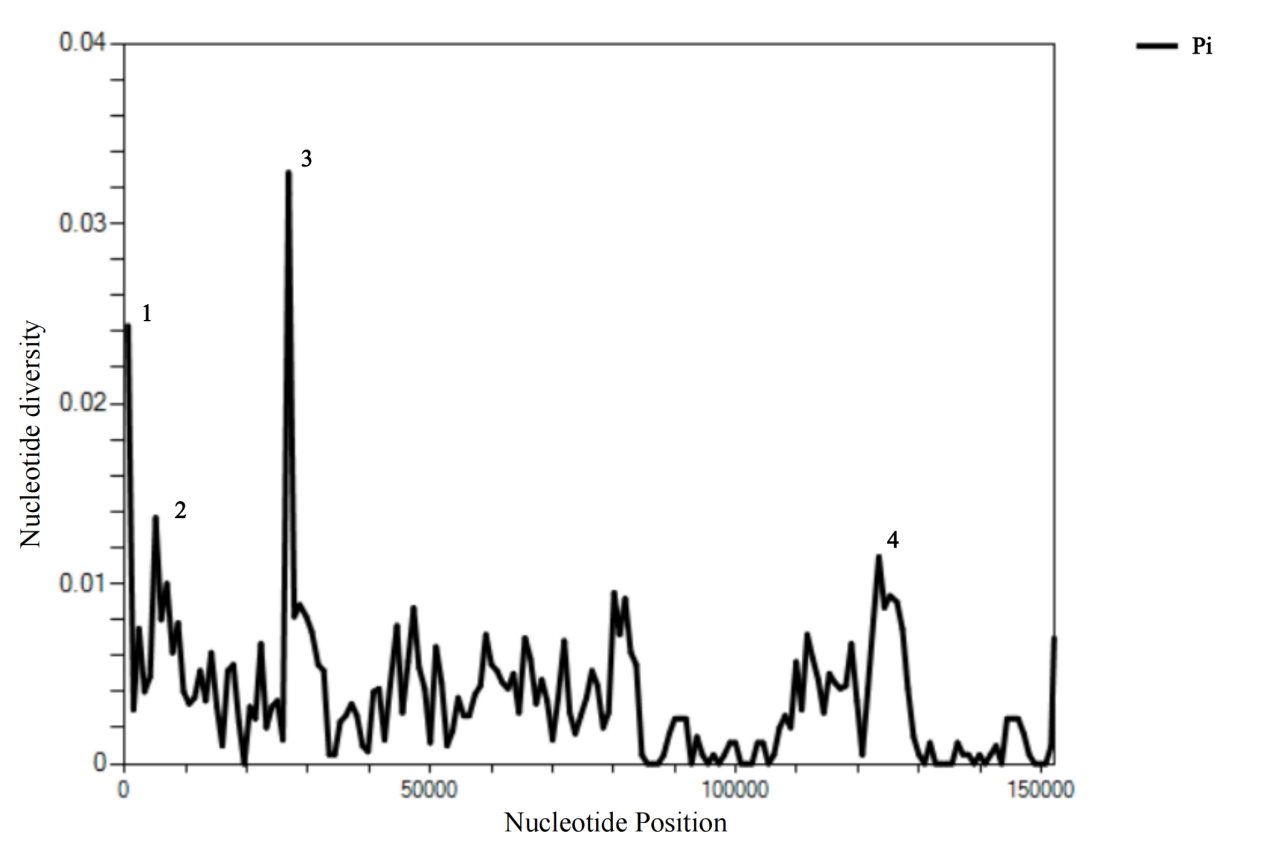
**Figure S3.** Detection of highly variable regions with DnaSP based on complete chloroplast genome sequences from *Primula amethystina* subsp. *argutidens* and 3 other *Sect. Amethyatina* species of *Primula amethystina* (NC_053577), *Primula faberi* (NC_053576) and *Primula virginis* (NC_053581)*.* Sliding windows with a window length of 1000 bp and a step size of 900 bp. Pi values greater than 0.01 are considered highly variable regions.
